# Seroprevalence for Hepatitis E and Other Viral Hepatitides among Diverse Populations, Malawi

**DOI:** 10.3201/eid2107.141748

**Published:** 2015-07

**Authors:** Taha E. Taha, Laura K. Rusie, Alain Labrique, Mulinda Nyirenda, Dean Soko, Melvin Kamanga, Johnstone Kumwenda, Homayoon Farazadegan, Kenrad Nelson, Newton Kumwenda

**Affiliations:** Johns Hopkins University, Baltimore, Maryland, USA (T.E. Taha, L.K. Rusie, A. Labrique, H. Farazadegan, K. Nelson, N. Kumwenda);; Queen Elizabeth Central Hospital, Blantyre, Malawi (M. Nyirenda);; Johns Hopkins Research Project, Blantyre (D. Soko, M. Kamanga);; University of Malawi, Blantyre (J. Kumwenda)

**Keywords:** Hepatitis, viruses, HEV, HAV, HBV, HCV, HIV, prevalence, Malawi

## Abstract

Data on prevalence of hepatitis E virus (HEV) in Malawi is limited. We tested blood samples from HIV-uninfected and -infected populations of women and men enrolled in research studies in Malawi during 1989–2008 to determine the seroprevalence of HEV, hepatitis A virus (HAV), hepatitis B virus (HBV), and hepatitis C virus (HCV). Samples were tested for IgG against HEV, total antibodies against HAV and HCV, and presence of HBV surface antigens. Of 800 samples tested, 16.5% were positive for HEV IgG, 99.6% were positive for HAV antibodies, 7.5% were positive for HBV surface antigen, and 7.1% were positive for HCV antibodies. No clear trends over time were observed in the seroprevalence of HEV, and HIV status was not associated with hepatitis seroprevalence. These preliminary data suggest that the seroprevalence of HEV is high in Malawi; the clinical effects may be unrecognized or routinely misclassified.

## Abstract 2

Analysis of blood samples collected over 20 years suggests substantial underestimation of this virus in this country.

Hepatitis E virus (HEV) is primarily a waterborne virus that is transmitted by the fecal–oral route. First recognized in the early 1980s, it is now acknowledged to be the primary cause of enterically transmitted non-A, non-B hepatitis (*1*). HEV has 1 serotype and 4 genotypes (*1*). Clinical characterization of HEV infection is similar to that of other viral hepatitis infections, ranging from asymptomatic infection to fulminant hepatitis (*2*). Although illness caused by HEV most often tends to be mild and self-limiting, high rates of illness and death among pregnant women is a unique complication and key epidemiologic feature of HEV infection. Additionally, chronic infection leading to fibrosis and cirrhosis of the liver can occur in the immunosuppressed (*3*).

Currently, no data on HEV seroprevalence are available for Malawi. However, outbreaks of HEV infection have been documented in several countries in the southern and eastern regions of Africa. In Zambia, the overall seroprevalence of HEV was 42% among 106 adults who participated in a community study in 1999; among children who were included in a prospective study of the same community in 2011, the seroprevalence of HEV was 8% in age group 1–4 years (n = 96), 16% in age group 5–9 years (n = 62), and 36% in age group 10–14 years (n = 36) (*4*). In northern Uganda, surveillance of health care facilities during 2010–2012 showed that 42% of 347 persons with reported acute jaundice syndrome cases had hepatitis E, 14% had hepatitis B, and 5% had hepatitis C (*5*). During 2012 in a refugee camp in eastern Kenya, 77.1% of 170 samples from persons with acute jaundice syndrome were positive for HEV IgM, RNA, or both (*6*). Data from earlier studies in Tanzania suggested either lack of exposure or low levels of HEV among women (*7**,**8*). A review of the epidemiology of HEV in Africa by Kim et al. (*9*) provides a listing of seroprevalence of HEV antibodies in various African countries.

Similar to HEV, HAV is transmitted by the fecal–oral route, although the epidemiology of the viruses is substantially different. Infection with HAV is considered a childhood disease in developing countries; nearly all children are infected at an early age. Disease tends to be mild in children and does not result in chronic infection (*10*). Unlike HAV and HEV, hepatitis B and C viruses (HBV and HCV) are transmitted through contact with infectious body fluids and can cause acute or chronic infection. Acute infection with HBV or HCV can manifest with a wide range of mild to severe symptoms. Chronic HBV and HCV infection can lead to serious outcomes such as cirrhosis, cancer, and failure of the liver (*11**, **12*). High HBV and HCV prevalence have been reported in southern Africa, where HIV prevalence is also high (*13*). HCV prevalence in Africa varies by country; estimates range from 1% to 10% (*14*). However, it is unclear whether HCV seroprevalence on the basis of antibody testing alone represents a true estimate because a high number of false-reactive results (compared to those for HCV RNA) have been reported in several HIV-prevalent populations in Africa (*15**,**16*). The rate of chronic HBV carriers in sub-Saharan Africa is estimated to be >8% (*17*). 

Previous studies have shown that HBV and HCV are prevalent in Malawi. Among patients in hospitals in Malawi, 17.5% tested positive for hepatitis B surface antigen (HBsAg), and samples from 4.5% were HCV antibody–positive (*18*). Among male sugar estate workers in Malawi, 14.9% tested positive for HBsAg, and samples from 10.6% were HCV antibody–positive (*19*).

Co-infection of HIV and HBV or HCV leads to accelerated progression of liver disease (*13*). The interaction of HEV with HIV has not yet been confirmed but is conceivable, and a strong association has been reported among adults in a retrospective study in Zambia in which 28% of HIV-seronegative and 71% of HIV-seropositive adults were found to be HEV seropositive (*4*). Superinfection with >1 type of hepatitis has been shown to cause severe disease. Children with simultaneous infection of HAV and HEV may experience accelerated disease progression. A study among persons who chronically carried the HBV surface antigen showed rapid clinical deterioration when co-infected with HEV (*20*). The high prevalence of HIV in Malawi, combined with the severe implications of co-infections with hepatitis viruses, necessitate clarification of the levels of multiple hepatitis virus infections within the same population. The primary aim of this study, conducted in 2012, was to determine the seroprevalence of HEV, as well as HAV, HBV, and HCV, in samples collected during 1989–2008 from diverse adult populations in Malawi.

## Methods

### Study Design and Populations

In a cross-sectional study, we analyzed serum and plasma samples collected during 6 studies in Malawi (Table 1) (*21**–**26*). These studies were conducted during 1989–2008 and represent diverse populations of HIV-infected and HIV-uninfected adult men and women from rural and urban settings in Malawi. Almost all HIV-infected persons in these studies were antiretroviral naive. Selection of samples stored at –80°C for laboratory testing was aimed to include approximately equal numbers of HIV-negative and HIV-positive samples from each year, on the basis of availability of sufficient sample volume, to test for all hepatitides. Inclusion of the samples was not based on previous knowledge of demographic factors of the study populations. In addition to collecting samples, we used structured case report forms to document demographic and clinical data.

**Table 1:** Description of studies included in analysis of hepatitis virus seroprevalence, Malawi, 1989–2008*

Study name, period (reference)	Study site/setting	Study type	Study population	HIV status	Aims of study
ICAR,1989–1994 (*21*,*22*)	Queen Elizabeth Central Hospital, Blantyre/urban	Observational cohort	Pregnant and postpartum women	+/–	Assess adverse pregnancy outcomes and rates of mother-to-child transmission of HIV
SUCOMA, 1998 (*23*)	Nchalo District, sugar estate/rural, occupational	Observational survey	Adult men	+/–	Identify epidemiologic and biologic determinants of HIV infection in an occupational male cohort
NVAZ, 2000–2004 (*24*)	Queen Elizabeth Central Hospital, Blantyre/urban	Randomized clinical trial	Pregnant women	+	Assess efficacy of short antiretroviral post-exposure prophylaxis to prevent mother to child transmission of HIV
MWANZA, 2001 (T.E. Taha, unpub. data)	Mwanza District/rural	Observational survey	Adult men and women	+/–	Assess risk factors associated with prevalent HIV infection in a rural area adjacent to the borders with Mozambique
METRO, 2003–2005 (*25*)	Queen Elizabeth Central Hospital; health centers in Blantyre/urban	Randomized clinical trial	Adult women (nonpregnant)	+/–	Assess the efficacy of intermittent intravaginal metronidazole gel use in reducing bacterial vaginosis infection
PEPI, 2004–2008 (*26*)	Queen Elizabeth Central Hospital; health centers in Blantyre/urban	Randomized clinical trial	Pregnant women	+	Assess efficacy of extended antiretroviral postexposure prophylaxis to prevent mother to child transmission of HIV

*ICAR, International Collaborations on AIDS Research; SUCOMA, a study of prevalence among male workers for the Sugar Company of Malawi; NVAZ, a study of efficacy of nevirapine/zidovudinel postexposure prophylaxis to prevent mother-to-child transmission of HIV; MWANZA, a study of risk factors associated with prevalent HIV infection in the rural town of Malawi; METRO, a study in which efficacy of intravaginal metronidazole gel in reducing bacterial vaginosis was assessed; PEPI, a study of antiretroviral postexposure prophylaxis to prevent mother to child transmission of HIV.

All participants signed written informed consent and agreed to provide study samples. All studies were approved by appropriate institutional review boards in the United States and Malawi. 

### Laboratory Tests for Hepatitis Seroprevalence

#### HEV Testing

Serum or plasma samples were tested for the presence of antibodies against HEV by using Wantai HEV-IgG ELISA kits (Wantai Biologic Pharmacy Enterprise Co., Ltd., Beijing, China). The Wantai assay uses a recombinant capsid protein (E2) encoded by the conserved region of open reading frame 2 of HEV. Compared with the Genelabs HEV IgG EIA (Genelabs, Inc., Singapore), the Wantai assay has been shown to be more sensitive (*27*). The HEV assays were performed manually following the manufacturer’s instructions in the serology laboratory of the Department of Epidemiology at Johns Hopkins Bloomberg School of Public Health in Baltimore, Maryland, USA.

#### Testing for Other Hepatitides

We used the same samples tested for HEV to additionally test for total antibodies (IgG and IgM) against HAV and HCV and for the presence of HBsAg by using commercially available kits from Bio-Rad Laboratories (Hercules, CA, USA). The HAV kits detect both acute and past infection; the HCV assays detect acute, past, and chronic infections; and the HBsAg assay detects acute and chronic infection with HBV. These tests were performed by using the automated EVOLIS microplate system from Bio-Rad at the Johns Hopkins Bloomberg School of Public Health in Baltimore. The Johns Hopkins School of Public Health serology laboratory is Clinical Laboratory Improvement Amendments–certified to perform virologic and serologic testing.

Because of operational constraints, all samples were tested once for each type of hepatitis and no duplicate testing was done. Quality control was performed with the inclusion of positive and negative controls in each assay. Results were considered positive when the optical density of a well was equal to or greater than the cutoff value calculated for each run of each assay as recommended by the manufacturer.

#### Statistical Analysis

Overall seropositivity for each virus was estimated and stratified by study year, HIV status, sex, and age category. Associations of the 4 hepatitides with selected exposure variables were assessed by using logistic regression. The covariates considered were education level, rural or urban residence, marital status, employment, running water in house, electricity in house, parity, multiple sexual partners, reported history of sexually transmitted infections, and condom use. Variables with a statistically significant association with a hepatitis seroprevalence in univariate analysis as well as variables considered epidemiologically important were included in the multivariate logistic regression to estimate adjusted odds ratios. Because of differences in follow-up periods and inconsistencies in availability of comparable data, we used baseline data for all covariates, regardless of whether an enrollment or follow-up sample was used for the hepatitis testing. A p value of ≤0.05 was considered statistically significant. Analysis was performed by using Stata version 11.2 (StataCorp LLC, College Station, TX, USA).

Previous data on the prevalence of hepatitis E in Malawi was not available. To estimate an appropriate sample size for this analysis, a conservative prediction of 2% prevalence was used. A sample size of 800 was determined to enable detection of a prevalence of 2% with a precision of 1% (*28*).

## Results

Table 2 shows the distribution of hepatitis seropositivity overall and across main exposure variables. Overall, 132 (16.5%) of 800 samples tested positive for HEV antibodies. The overall seroprevalence of HAV antibodies, HBsAg and HCV antibodies in samples collected from different populations in Malawi was 99.6%, 7.5%, and 7.1%, respectively (Table 2). HEV seroprevalence was higher among HIV-uninfected (20.2%) than among HIV-infected (12.9%) persons, and HCV seroprevalence was higher among male (10.2%) than female (5.6%) (p<0.05 by Fisher exact test) participants. Seroprevalence varied by study year and age group; seroprevalence of enteric HEV was 20.8% for a male occupational cohort in the 1994–1999 Sugar Company of Mulawi (SUCOMA) study(*23*), and 26.4% for an female urban cohort in the 2003–2005 metronidazole gel (METRO) study (*25*) in which efficacy of intravaginal metronidazole gel in reducing bacterial vaginosis was assessed. These overall differences by study year were statistically significant (p<0.001 by χ^2^ test).

**Table 2:** Prevalence of anti-HEV IgG, total anti-HAV Ig, HBsAg, and total anti-HCV Ig in Malawi*

Characteristic	HEV			HAV			HBsAg			HCV	
	No. positive/no. tested	% Positive (95% CI)		No. positive/no. tested	% Positive (95% CI)		No. positive/no. tested	% Positive (95% CI)		No. positive/no. tested	% Positive (95% CI)
Total cohort	132/800	16.5 (13.9–19.1)		777/780	99.6 (99.2–100.0)		58/773	7.5 (5.6–9.4)		55/779	7.1 (5.3–8.9)
HIV status											
Negative	80/397	20.2 (16.2–24.1)		390/391	99.7 (99.2–100.0)		32/388	8.2 (5.5–11.0)		29/394	7.4 (4.8–10.0)
Positive	52/403	12.9 (9.6–16.2)		387/389	99.5 (98.8–100.0)		26/385	6.8 (4.2–9.3)		26/385	6.8 (4.2–9.3)
Sex											
M	45/246	18.3 (13.4–23.2)		244/245	99.6 (98.8–100.0)		25/245	10.2 (6.4–14.0)		25/245	10.2 (6.4–14.0)
F	87/554	15.7 (12.7–18.7)		533/535	99.6 (99.1–100.0)		33/528	6.3 (4.2–8.3)		30/534	5.6 (3.7–7.6)
Study, period											
ICAR, 1989–1995	6/70	8.6 (1.8–15.3)		70/70	100.0		0/70	0.0		2/70	2.9 (−1.1–6.9)
SUCOMA, 1994–1999	37/178	20.8 (14.8–26.8)		176/177	99.4 (98.3–100.0)		21/178	11.8 (7.0–16.6)		19/178	10.7 (6.1–15.3)
NVAZ, 2000–2003	19/165	11.5 (6.6–16.4)		157/157	100.0		6/156	3.9 (0.8–6.9)		8/148	5.4 (1.7–9.1)
MWANZA, 2001	13/100	13.0 (6.3–19.7)		99/99	100.0		7/98	7.1 (2.0–12.3)		9/99	9.1 (3.3–14.9)
METRO, 2003–2005	39/148	26.4 (19.2–33.5)		141/142	99.3 (97.9–100.0)		8/137	5.8 (1.9–9.8)		9/146	6.2 (2.2–10.1)
PEPI, 2004–2009	18/139	12.9 (7.3–18.6)		134/135	99.3 (97.8–100.0)		16/134	11.9 (6.4–17.5)		8/138	5.8 (1.8–9.7)
Age range, y											
15–19	15/77	19.5 (10.4–28.5)		75/75	100.0		5/76	6.6 (0.9–12.3)		6/75	8.0 (1.7–14.3)
20–29	72/438	16.4 (13.0–19.9)		424/426	99.5 (98.9–100.0)		33/420	7.9 (5.3–10.4)		30/424	7.1 (4.6–9.5)
30–39	28/191	14.7 (9.6–19.7)		185/186	99.5 (98.4–100.0)		12/184	6.5 (2.9–10.1)		12/186	6.5 (2.9–10.0)
40–49	7/60	11.7 (3.3–20.0)		59/59	100.0		3/59	5.1 (−0.7 to 10.9)		3/60	5.0 (−0.7 to 10.7)
50–59	6/18	33.3 (9.2–57.5)		18/18	100.0		2/18	11.1 (−5.0 to 27.2)		2/18	11.1 (−5.0 to 27.2)
60–69	2/4	50.0 (−41.9 to 141.9)		4/4	100.0		4/4	100.0		1/4	25.0 (−54.6 to 104.6)

*HEV, hepatitis E virus; HAV, hepatitis A virus; HBsAg, hepatitis B surface antigen; HCV, hepatitis C virus; ICAR, International Collaborations on AIDS Research; SUCOMA, a study of HIV prevalence among male workers for the Sugar Company of Malawi; NVAZ, a study of efficacy of nevirapine/zidovudinel postexposure prophylaxis to prevent mother-to-child transmission of HIV; MWANZA, a study of risk factors associated with prevalent HIV infection in the rural town of Malawi; METRO, a study in which efficacy of intravaginal metronidazole gel in reducing bacterial vaginosis was assessed; PEPI, a study of antiretroviral postexposure prophylaxis to prevent mother to child transmission of HIV.

Unlike HEV seroprevalence, the seroprevalence of the other enterically transmitted infection (HAV) did not show variation by year of study and was ≈100% in all studies. The prevalence of HBsAg and HCV antibodies was low (0% HBsAg; 2.9% HCV antibodies) in the 1989–1995 cohort of urban pregnant and postpartum women of the International Collaborations on AIDS Research (ICAR) study (*21**,**22*). In subsequent years, the reported prevalence of HBsAg and HCV antibodies increased and was approximately comparable in all studies reviewed for this study; exceptions are the high seroprevalence of both hepatitides in the men of the occupational cohort of SUCOMA and the high seroprevalence of HBsAg among women in the postexposure prophylaxis of infants (PEPI) cohort (2004–2009) (*26*). Stratification by age group (excluding those 60–69 years of age because of limited numbers) showed comparable HEV seroprevalence among persons <50 years of age (or trend of decrease with increase in age) and a seroprevalence of 33% among persons 50–59 years of age. The seroprevalence of HAV, however, was uniformly high and was ≈100% in all age groups. Both HBsAg and HCV seroprevalence were stable and comparable in age groups <50 (no. persons in older age groups was small). Among 757 persons with laboratory results available for both HBsAg and HCV, 7 were dually seropositive for HBsAg and HCV (0.9%; 95% CI 0.2–1.6; p = 0.11 by χ^2^ test); all 7 were HIV-uninfected.

Table 3 shows univariate (crude) and multivariate (adjusted) odds ratios (OR) of being seropositive for HEV. In the univariate analysis, participants in the SUCOMA (1994–1999) and METRO (2003–2005) studies were significantly more likely to be positive for HEV antibodies compared with participants in the ICAR earlier study (1989–1994) (OR 2.80 and 3.82, p = 0.03 and 0.004, respectively). In the multivariate analysis, only the METRO study year (2003–2005) remained statistically significant after adjusting for other variables included in Table 3 (p = 0.01 for METRO study). Although not statistically significant in the multivariate analysis, participants who tested HIV-positive and those who reported having running water in the house were less likely to be seropositive for HEV antibodies in the univariate analyses (HIV-positive OR 0.59, p = 0.01; have running water OR 0.61, p = 0.06).

**Table 3:** Crude and adjusted odds ratios for enterically transmitted hepatitis E virus, Malawi*

Characteristics	Crude OR	p value	Adjusted OR (95% CI)
Study, period			
ICAR, 1989–1995	Reference	NA	Reference
SUCOMA, 1994–1999	2.80	0.03	3.54 (0.75–16.76)
NVAZ, 2000–2003	1.39	0.51	1.59 (0.55–4.62)
MWANZA, 2001	1.59	0.37	1.85 (0.51–6.70)
METRO, 2003–2005	3.82	0.004	3.44 (1.37–8.67)
PEPI, 2004–2009	1.59	0.35	1.87 (0.64–5.49)
Enrollment year†	1.02	0.38	NA
HIV status			
HIV-negative	Reference	NA	Reference
HIV-positive	0.59	0.01	0.72 (0.38–1.36)
Age, y†	1.01	0.56	1.00 (0.97–1.02)
Age range, y			
15–19	Reference	NA	NA
20–29	0.81	0.51	NA
30–39	0.71	0.33	NA
40–49	0.55	0.22	NA
50–59	2.07	0.21	NA
60–69	4.13	0.17	NA
Sex			
M	Reference	NA	1.00
F	0.83	0.36	1.40 (0.41–4.76)
Setting			
Rural	Reference	NA	NA
Urban	0.85	0.41	NA
Running water in house			
No	Reference	NA	1.00
Yes	0.61	0.06	1.40 (0.41–4.76)
Electricity in house			
No	Reference	NA	NA
Yes	0.82	0.40	NA
Education			
None	Reference	NA	NA
Any	0.92	0.78	NA
Marital status			
Married	Reference	NA	NA
Single	0.60	0.14	NA
Employment status			
Unemployed	Reference	NA	NA
Employed	1.18	0.41	NA
Parity†	0.90	0.61	NA

*OR, odds ratio; ICAR, International Collaborations on AIDS Research; SUCOMA, a study of HIV prevalence among male workers for the Sugar Company of Malawi; NVAZ, a study of efficacy of nevirapine/zidovudinel post-exposure prophylaxis to prevent mother-to-child transmission of HIV; MWANZA, a study of risk factors associated with prevalent HIV infection in the rural town of Malawi; METRO, a study in which efficacy of intravaginal metronidazole gel in reducing bacterial vaginosis was assessed; PEPI, a study of antiretroviral postexposure prophylaxis to prevent mother-to-child transmission of HIV; NA: not applicable. †Continuous variable.

HAV seroprevalence was high, and the distribution was comparable across all variables and strata of each covariate; therefore, no associations of HAV with covariates included in this study were observed in both univariate and multivariate analyses. Table 4 shows the univariate, unadjusted ORs for HBsAg and HCV. Participants in the Nevirapine-Zidovudine (NVAZ) study were less likely to be positive for HBsAg than participants in the ICAR study (OR 0.30, p = 0.01 [*21,22*]). Residents of urban areas also had lower odds for testing positive for HBsAg compared with residents of rural areas (OR 0.57, p = 0.04), and female participants were less likely to test positive than male participants (OR 0.59, p = 0.05). A statistically nonsignificant trend of increasing odds of HBsAg over time on the basis of enrollment calendar year in the parent study is also shown in Table 4 (OR 1.07, p = 0.06). The univariate analysis of HCV association with the risk factors listed in Table 3 showed that female participants had statistically significantly lower odds of being seropositive for HCV compared with male participants, and urban residents were less likely to test positive than rural residents (OR 0.52 and 0.51, p = 0.02 and 0.02, respectively). Although not statistically significant (p = 0.06), the ORs for participation in the SUCOMA study and having electricity in the house (an indicator of higher socioeconomic status) were 4.06 (95% CI 0.92–17.93) and 0.46 (95% CI 0.20–1.03), respectively. In the multivariate logistic regression analyses for the association of HBsAg and HCV with various covariates (study enrollment, HIV status, age, sex, whether there were multiple lifetime sexual partners, history of sexually transmitted diseases, and history of condom use), only enrollment in the NVAZ study (2000–2003) remained significantly associated with lower likelihood to test positive for HBsAg (adjusted OR 0.24, p = 0.006). No other variables were found to be associated with HBV or HCV seroprevalence.

**Table 4:** Crude odds ratios for the association of HBV and HCV with selected exposures in studies of hepatitis prevalence, Malawi, 1989–2009*

Characteristics	HBsAg			HCV	
	OR	p value		OR	p value
Study, period					
ICAR, 1989–1995	Reference	NA		Reference	NA
SUCOMA, 1994–1999	0.99	0.97		4.06	0.06
NVAZ, 2000–2003	0.30	0.01		1.94	0.41
MWANZA, 2001	0.57	0.23		3.40	0.13
METRO, 2003–2005	0.46	0.08		2.23	0.31
PEPI, 2004–2009	ND	ND		2.09	0.36
Enrollment year, continuous	1.07	0.06		1.00	0.96
HIV status					
HIV-negative	Reference	NA		Reference	NA
HIV-positive	0.81	0.43		0.91	0.74
Age, continuous, years	0.99	0.68		1.00	0.79
Age range, y					
15–19	Reference	NA		Reference	NA
20–29	1.21	0.70		0.88	0.78
30–39	0.99	0.99		0.79	0.66
40–49	0.76	0.72		0.61	0.49
50–59	1.78	0.52		1.44	0.67
60–69	ND	ND		3.83	0.28
Sex					
M	Reference	NA		Reference	NA
F	0.59	0.05		0.52	0.02
Setting					
Rural	Reference	NA		Reference	NA
Urban	0.57	0.04		0.51	0.02
Running water in house					
No	Reference	NA		Reference	NA
Yes	1.00	1.00		0.74	0.43
Electricity in house					
No	Reference	NA		Reference	NA
Yes	1.17	0.61		0.46	0.06
Education					
None	Reference	NA		Reference	NA
Any	0.72	0.50		0.93	0.88
Marital status					
Married	Reference	NA		Reference	NA
Single	1.90	0.07		0.42	0.15
Employment					
Unemployed	Reference	NA		Reference	NA
Employed	1.61	0.09		1.45	0.19
Parity (continuous)	0.86	0.20		0.80	0.09
Lifetime sex partners					
≤1	Reference	NA		Reference	NA
>1	1.29	0.37		1.19	0.54
History of STIs					
Never	Reference	NA		Reference	NA
Ever	0.63	0.29		0.79	0.57
Condom use					
Never	Reference	NA		Reference	NA
Ever	0.75	0.38		0.95	0.88
*HBsAg, hepatitis B surface antigen; HCV, hepatitis C virus; OR, odds ratio; ICAR, International Collaborations on AIDS Research; SUCOMA, a study of HIV prevalence among male workers for the Sugar Company of Malawi; NVAZ, a study of efficacy of nevirapine/zidovudinel post-exposure prophylaxis to prevent mother-to-child transmission of HIV; MWANZA, a study of risk factors associated with prevalent HIV infection in the rural town of Malawi; METRO, a study in which efficacy of intravaginal metronidazole gel in reducing bacterial vaginosis was assessed; PEPI, a study of antiretroviral postexposure prophylaxis to prevent mother to child transmission of HIV; NA, not applicable; ND; no data; STI, sexually transmitted infection.					

## Discussion

This study provides preliminary estimates of HEV seroprevalence in Malawi, which has a population of ≈12 million, is mostly rural, and has a limited safe water supply and constrained health care services (*29*). Overall, 16.5% (95% CI 13.9%–19.1%) of the samples were positive for HEV IgG. In addition to examining seroprevalence of HEV in Malawi, this study has several other notable features: 1) samples from 6 epidemiologic studies conducted by the same research team among adult men and women in urban and rural Malawi during 1989–2008 were included, representing diverse populations and time periods; 2) analyses of viral hepatitides seroprevalence included both HIV-negative and HIV-positive persons (the prevalence of HIV remains high in southern Africa); and 3) these samples were also tested for total antibodies (IgG and IgM) against HAV and HCV and for the presence of HBsAg, leading to a comprehensive seroprevalence profile of all viral hepatitides in these populations. 

Although some statistically significant differences were observed in univariate analyses for the association of selected risk factors with seroprevalence of HEV, none of these remained significant in the multivariate logistic regression analyses, including HIV status, age category, sex, or having access to running water in the household. Recent studies have also found no differences in infection rates by gender or HIV status (*1**,**30*). The current study did not include children, which likely explains why previously identified trends of increasing infection levels with age were not detected. We also did not find a trend of seroprevalence over time. The only risk factor substantially associated with HEV seroprevalence was the METRO study period, 2003–2005, as compared to the ICAR study, which was conducted during 1989–1995. Both studies were conducted among women in Blantyre, Malawi. 

It is unclear what may have caused the METRO study participants to have higher levels of HEV seroprevalence. We did not collect data on seasonality and source of water and food; over the years Malawi had fluctuating bouts of drought and food scarcity. We tested diverse study populations from rural and urban settings recruited over a period of nearly 20 years, but no clear major trends were observed.

As expected, HAV seroprevalence was nearly universal in this sample of adults, with an overall prevalence of ≈100%. In low-resource settings such as Malawi, HAV is a childhood infection, and nearly all persons are infected within the first few years of life. Though children were not included in our study, it should be considered that they can be exposed to HEV because of the high prevalence among adults we observed. Although HAV and HEV infection tend to cause mild disease independently, concurrent infections with these pathogens in children may lead to accelerated disease progression (*20*). The changing epidemiology of HAV in some settings related to rapid industrialization and urban migration in developing countries may result in some children not being exposed to HAV (*10*).

An overall prevalence of 7.5% (95% CI 5.6%–9.4%) was found for HBsAg and 7.1% (95% CI 5.3%–8.9%) for HCV. The results of the HBsAg and HCV seroprevalence tests are consistent with previously published data from sub-Saharan Africa. Specifically in Malawi, a study published in 2002 reported a prevalence of 14.9% for HBsAg and 10.6% for HCV among the SUCOMA participants (*23*). In our study, we found a prevalence of 11.8% (95% CI 7.0%–16.6%) and 10.7% (95% CI 6.1%–15.3%) for HBsAg and HCV, respectively, in the SUCOMA study samples. Women who enrolled in NVAZ had lower odds of testing positive for HBsAg compared to those in ICAR. The only distinct difference between these studies was the HIV status of the women (some women in the ICAR study were HIV negative), but because this was included in the multivariate logistic regression, results should not be confounded by the HIV status. No covariates were found to be associated with HCV seroprevalence in this study.

HEV epidemiology is evolving, and circulating genotypes and modes of transmission appear to be complex in both developing and industrialized countries (*1*). We do not know in Malawi if only waterborne HEV genotypes are the source of potential infections or if other less virulent zoonotic HEV genotypes coexist. High seroprevalence of HEV antibodies does not imply clinical infection or increased association with clinical complications. Nonetheless, some misclassification of acute viral hepatitis is likely in Malawi where screening for HEV antibodies is not performed. Because the seroprevalence of HEV in this study was twice that of HBV or HCV, cases of acute hepatitis may frequently be caused by HEV as opposed to HBV or HCV. Notably, 15.7% (95% CI 12.7%–18.7%) of women in this study cohort had antibodies against HEV, which can pose serious health risks for pregnant women (*3*). 

Although no association was found that HIV-positive persons are at higher risk for anti-HEV than are HIV-negative persons, the finding that 12.9% (95% CI 9.6%–16.2%) of samples from HIV-positive persons were HEV positive is of concern. Malawi and other countries in sub-Saharan Africa have high levels of HIV infection, and co-infection with HEV may lead to chronic HEV infection and accelerated disease progression.

The findings from this study should be regarded as preliminary and require confirmation. Therefore, additional epidemiologic and virological studies should be conducted in this region. As with all cross-sectional data, inferences regarding associations should be interpreted with caution. A related limitation is that the samples used for serologic testing in this study included both enrollment and follow-up samples (in cohort studies) to maximize availability of samples, whereas the covariate data used for all participants was baseline enrollment data. Despite these limitations, we suspect that many of the covariates used, such as having running water in the house, did not substantially change over time. Very few risk factors associated with HEV, HBV, or HCV seroprevalence were found. This result may be because of differences in population characteristics or definitions used in multiple studies. For example, participants in the SUCOMA study were all men working in a rural sugar estate occupational setting and may be considered to be at high risk (*23*). We also did not have data on some behaviors associated with HCV seroprevalence, such as intravenous drug use, although the practice is very rare in Malawi. The lack of association between viral hepatitides and various risk factors conventionally collected in these studies suggests that better data collection tools to evaluate potential risk factors and different study designs targeting at risk populations may need to be considered in future studies.

Confirmation of the hepatitis testing results, particularly HEV seroprevalence, reported in this study will be critical in subsequent studies because each sample was tested once. Retesting of a subset of positive and negative samples should ideally be done with the same Wantai assay used in this study as well as with other assays. The hepatitis A, B, and C assays used were commercially available in the United States and well validated, but it is recommended that samples be tested in duplicate for these assays. The consistency of our results with findings from previous seroprevalence studies in Malawi and other countries suggests misclassification may be minimal (taking into account the controversy regarding HCV testing). Molecular analysis to determine what genotypes of HEV are prevalent in Malawi may also be useful and would provide further insight into the epidemiology of this virus.
